# A MLR-Based Approach to Analyze Regulators of T Lymphocyte Activation In Vivo

**DOI:** 10.3390/ijms23105337

**Published:** 2022-05-10

**Authors:** Jiří Koutník, Victoria Klepsch, Maria Pommermayr, Nikolaus Thuille, Gottfried Baier, Kerstin Siegmund

**Affiliations:** Institute of Cell Genetics, Medical University Innsbruck, 6020 Innsbruck, Austria; jiri.koutnik@i-med.ac.at (J.K.); victoria.klepsch@i-med.ac.at (V.K.); maria.pommermayr@i-med.ac.at (M.P.); nicolaus.thuille@i-med.ac.at (N.T.); gottfried.baier@i-med.ac.at (G.B.)

**Keywords:** in vivo model, T cell activation, allogenic response, MLR, GVHD model

## Abstract

Depending on the context, robust and durable T lymphocyte activation is either desirable, as in the case of anti-tumor responses, or unwanted, in cases of autoimmunity when chronic stimulation leads to self-tissue damage. Therefore, reliable in vivo models are of great importance to identify and validate regulatory pathways of T lymphocyte activation. Here, we describe an in vivo mixed-lymphocyte-reaction (MLR) approach, which is based on the so-called parent-into-F1 (P → F1) mouse model in combination with the congenic marker CD45.1/2 and cell proliferation dye-labeling. This setup allows us to track adoptively transferred allogenic CD4^+^ and CD8^+^ T lymphocytes and analyze their phenotype as well as the proliferation by flow cytometry in the blood and spleen. We could show hypo-reactive responses of T lymphocytes isolated from knockout mice with a known defect in T lymphocyte activation. Thus, this MLR-based in vivo model provides the opportunity to analyze positive regulators of T cell responses under physiological conditions of polyclonal T lymphocyte activation in vivo.

## 1. Introduction

The importance of a finely balanced T cell response—strong enough to prevent cancer and to protect from infectious diseases while not damaging its own tissue—is reflected in the increasing amount of therapeutic approaches targeting T cells. These strategies aim to either suppress T cell activity, as in the case of autoimmunity and organ transplantation, or boost it, which is preferable for anti-tumor approaches. The latter applies, for example, for an adoptive transfer of T cells (ACT) expressing a chimeric antigen receptor as a therapeutic tool. In this regard, it is of great interest to understand the molecular switches of T cell activation, to modulate their responsiveness and the T cell-mediated immune response outcome. Thus, there is a strong need for physiological model systems to test potential modulators of T cell responses.

However, since T cells demonstrate an enormous range (estimated about 10^6^–10^8^, [1]) of distinct T cell receptors (TCR), finding appropriate tools for investigations is still challenging. In vitro, the issues of unknown antigen-specificity and low precursor frequency can be circumvented by polyclonal stimulation, using, for example, antibodies targeting the TCR-associated CD3ε-chain and the CD28 co-receptor, phorbol esters and ionomycin, which act intracellularly to activate the signaling cascade downstream of the TCR, or mitogens, such as concanavalin A. Nonetheless, these strong stimuli are quite artificial and therefore not truly reflecting the physiological stimulation through the TCR via peptide-loaded MHCs (major histocompatibility complex) on cells that additionally provide signals by co-stimulatory receptors or secreted cytokines. One step towards a more physiological in vitro T cell activation approach can be reached by a so-called allogenic stimulation. Allogenicity was first noted in the 1950s, when George Snell bred two mouse strains, which were nearly identical but differing in tissue compatibility upon transplantation [2]. Jean Dausset observed an immune response against donor leukocytes during human blood transfusion [3], which later on led to the discovery of the MHC locus. Briefly, in an allogenic setting, T cells confound a non-self MHC donor peptide complex with a self-MHC, presenting a foreign peptide. This leads then to an erroneous T cell activation, even in the absence of any signs of danger. Besides the significance of this process for transplantation medicine, the system can also be hijacked for T cell studies using in vitro mixed lymphocyte reactions (MLR). In this setting, T cells of interest are mixed with at least 3-fold more murine splenocytes or peripheral blood mononuclear cells (PBMCs) of a different MHC haplotype [4,5]. To avoid proliferation of the stimulator splenocytes, these are either irradiated or mitomycin C-treated. However, these treatments lead to many dead cells within the co-culture that might influence the responder T cells’ behavior. Alternatively, a “one-way MLR” can be applied that uses for stimulation much less allogenic dendritic cells, which are matured by LPS treatment but are not treated in any other way, leading to cell cycle arrest and thus cell death.

Although providing stimulation through the TCR, all these in vitro models face the problem of not taking into account migration and localization within tissues, representing an environment lacking the stimulatory or inhibitory factors provided by other cell types. This obstacle can only be overcome using in vivo models.

Thus, for example, mice can be studied upon immunization with aluminum hydroxide-emulsified ovalbumin (OVA-alum). However, the tracking of T cell activation using this in vivo approach is challenging due to the low frequency of (naïve) antigen-specific precursors (approx. 0.8 to 10 cells per million naïve CD4^+^ T cells; [6]). Thus, with this approach, only a small number of cells can be analyzed at the endpoint, which makes statements about activation/exhaustion difficult, even though antigen-specific T cells can be detected via MHC-tetramer technology. Moreover, using tetramers covers only the response to one specific peptide, which does not reflect the whole T cell response to the protein.

Other in vivo models are based on an ACT of TCR-transgenic (tgTCR) T cells, using a monoclonal TCR, followed by immunization with the cognate antigen. For instances, OT-I and OT-II mice are used, whose CD8^+^ and CD4^+^ T cells, respectively, harbor a TCR specific for a peptide from chicken ovalbumin [7,8]. In this way, the frequency of antigen-specific T cells is much higher and therefore easier to investigate, especially when antibodies specific for the tgTCR-T cells are available. However, when using this approach, one has to consider the drawbacks of this system. For example, tgTCR-T cells experience an artificially strong activation and demonstrate an altered thymic development; e.g., OT-I mice develop almost just CD8^+^ T cells due to MHC class I-mediated positive selection. Furthermore, no polyclonal response is analyzed using this approach.

Another possibility to address T cell responses in vivo is based on the above-described MLR approach, which increases the frequency of potential responders to approximately 7% [9]. Thereby, recipient mice, which are lymphodepleted or totally body irradiated to prevent rejection of the transferred cells, receive an allogenic T cell-depleted bone marrow transplantation (TCD-BMT) along with allogenic T cells. This setup is used as a MHC-mismatched mouse model of T cell-mediated acute graft-versus-host disease (aGVHD). Direct cytotoxic effects of donor T cells on recipient tissues lead to damage, and the release of pro-inflammatory cytokines contributes to severe immunopathology, which is usually lethal, at the latest, four weeks after transfer (reviewed in [10]).

Unlike the aGVHD model described above, the so-called parent-into-F1 (P → F1) model avoids rejection of the transferred T cells of a certain haplotype by using semi-allogenic F1-hybrids of this haplotype and of an allogenic haplotype as a host. For instance, C57BL/6 splenocytes or sorted T cells (MHC haplotype b) are transferred to offspring of a C57BL/6 × DBA2 breeding (b/d haplotype). Using this approach, the irradiation of the recipients is not necessary. The transferred parental strain T cells will respond vigorously to the mismatched MHC d haplotype of the recipient.

Here, we combined the P → F1 model with the congenic marker of CD45, which allows us to track the activation of transferred T cells on single cell level. Our analyses were performed within eight days after ACT before the recipient mice develop any obvious signs of aGVHD. Using this approach, we have additionally analyzed T cells derived from *PKCθ*-knockout mice. PKCθ is a central signaling molecule downstream of the TCR, activated by DAG-binding, whose deficiency is known to impair T cell activation resulting in reduced interleukin (IL)-2 expression and proliferation ([11,12]). Thus, we have validated the model for positive regulators of T cell activation.

## 2. Results

### 2.1. Characterization of an Allogenic In Vivo Model to Track T Cell Responses on a Single Cell Level

**Figure 1:**
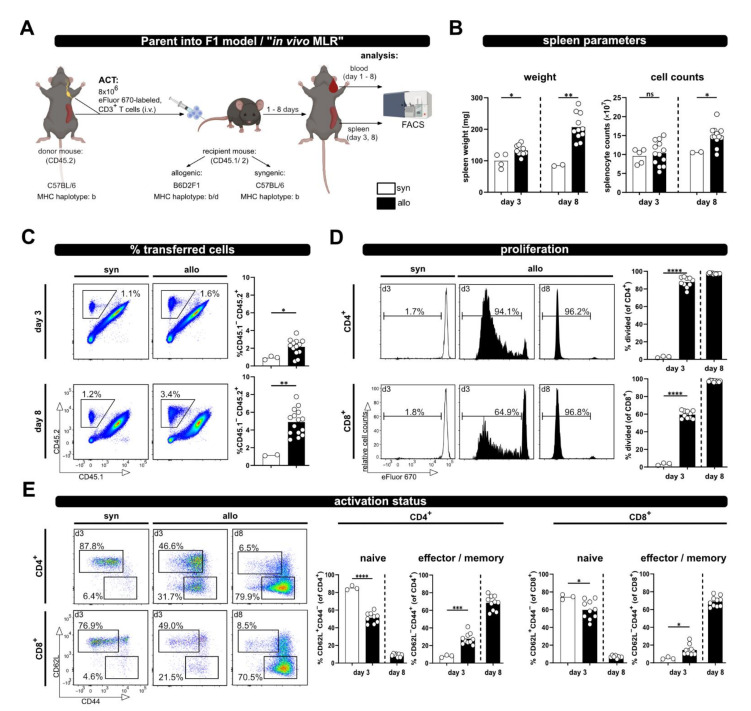
The P → F1 approach to analyze T cell activation in vivo in spleen and blood. (**A**) Experimental setup: 8 × 10^6^ wild type; eFluor670 proliferation dye-labeled CD3^+^ T cells were adoptively transferred i.v. to either syngenic or allogenic recipients. Transferred T cells were analyzed from spleen and blood of recipient mice by flow cytometry at the indicated time points. (**B**) Spleen weight and splenocyte counts at day 3 and 8 after ACT. (**C**) The frequency of transferred T cells (CD45.2^+^) in the spleens of allogenic or syngenic recipients (both CD45.1^+^/2^+^) was determined by flow cytometry using CD45.1 and CD45.2 specific antibodies. (**D**) T cell proliferation was analyzed based on dilution of the proliferation dye eFluor670 with each cell division. Proliferation of transferred CD4^+^ and CD8^+^ T cells is depicted as percent of divided cells. (**E**) The frequency of naive and effector/memory subsets among transferred CD4^+^ and CD8^+^ T cells was determined by CD44 and CD62L staining. Data are represented as individual values plus mean. Representative FACS dot plots or histograms of individual mice are shown. ns = not significant, * *p* < 0.05, ** *p* < 0.01, *** *p* < 0.001, **** *p* < 0.0001; unpaired *t*-test.

In order to investigate T cell activation upon allogenic stimulation, we have adapted the P → F1 model by the additional inclusion of a congenic marker. In brief, 8 × 10^6^ MACS-sorted CD3^+^ T cells from CD45.2^+^/2^+^ C57BL/6 mice, labeled with the proliferation dye eFluor670, were adoptively transferred by intravenous injection into syn- or allogenic recipients (Figure 1A). The latter were bred by crossing CD45.1^+^/1^+^ C57BL/6 females (MHC haplotype b) to CD45.2^+^/2^+^ DBA2 males (MHC haplotype d), resulting in CD45.1^+^/2^+^ B6D2F1 hybrids (MHC haplotype b/d). Syngenic CD45.1^+^/2^+^ C57BL/6 mice (haplotype b) were used as control recipients.

Based on the expression of the congenic marker CD45, the transferred T cells can be now identified by flow cytometry. Proliferation was analyzed by determining the dilution of the eFluor670 dye, which binds irreversibly to intracellular proteins and is equally distributed between two daughter cells. The analyses were performed on day 3, a rather early time point upon ACT, and at day 8 as a later time point; however, this was before any GVHD symptoms should occur [13]. Indeed, besides significantly enlarged spleens, especially on day 8, in allogenic compared to syngenic hosts (spleen weight, Figure 1B), we did not observe any health issues. Accordingly, splenocyte counts at day 8 in allogenic recipients were also significantly higher than in syngenic recipients at the same time point (Figure 1B).

Moreover, while the frequency of transferred T cells in the spleens of syngenic recipients remained constant over time, in allogenic recipients it was already significantly higher on day 3 and further increased on day 8 (average of 2.35 ± 0.98% at day 3 and 4.26 ± 1.26% at day 8; Figure 1C). Correspondingly, the vigorous proliferation of T cells in an allogenous environment was observed by dilution of the proliferation dye (Figure 1D). CD4^+^ T cells responded faster than CD8^+^ T cells, with more divided CD4^+^ than CD8^+^ T cells at day 3 after ACT, which is in accordance with the literature on the P → F1 model, describing that donor CD4^+^ T cells initiate the GVHD response [14]. However, later on CD8^+^ T cells catch up, and at day 8 after ACT they had undergone at least eight cell cycles so that “all proliferation dye label” has been lost, now showing the same fluorescence intensity as unlabeled cells. The different proliferation kinetic of CD4^+^ and CD8^+^ T cells is also evident by the CD4^+^ to CD8^+^ T cell ratio. Thus, CD4^+^ T cells reach a 5.5 ± 0.3 fold number of CD8^+^ T cells at day 3, but the ratio declines on average below one (0.9 ± 0.1) at day 8. Accordingly, T cell differentiation and acquisition of an effector/memory phenotype, visible by the loss of CD62L expression and upregulation of CD44, was observed in allogenic but not syngenic recipients (Figure 1E). In addition, this chronic stimulation, as it occurs in allogenic recipients, induced the up-regulation of inhibitory receptors (PD-1, Lag-3 and Tim-3), in particular at day 8 after ACT (Supplementary Figure S1).

**Figure 2:**
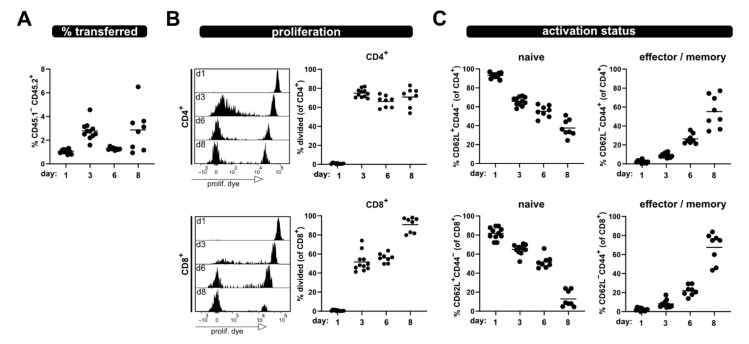
Kinetic of allogenic T cell activation in blood sample. Transferred T cells were analyzed from the blood at day 1, 3, 6 and 8 after transfer for (**A**) their frequency among lymphocytes, (**B**) proliferation of CD4^+^ and CD8^+^ T cells as well as (**C**) naive and effector/memory status—analogous to what is shown in Figure 1. Data are represented as individual values plus mean. Representative FACS histograms of individual mice are shown.

Furthermore, analysis of the same parameters was performed at several time points from blood; thus, allowing minimal-invasive kinetic analysis of T cell responses (Figure 2). A clear population of transferred T cells, which increased from day 1 to day 3, was detected in the blood samples. However, after this initial increase their frequency dropped at day 6, to then increase again at day 8. This phenomenon might be due to re-distribution. Nonetheless, especially evident in the blood kinetic samples are the distinct proliferation kinetics of CD4^+^ and CD8^+^ T cells (Figure 2B). Of note, a shift of the undivided eFluor670^high^ peak was observed over time due to protein turnover, which leads to reduced fluorescence intensity (histograms, Figure 2B).

However, a clear discrimination between divided and undivided T cells was still possible at all analysis time points. Additionally, analyses of the phenotype of the transferred T cells in the blood of allogenic recipients at several time points after ACT revealed that the frequency of naive T cells progressively declines, while the proportion of T cells with an effector phenotype increases (Figure 2C). At day 8, most of the transferred CD4^+^ and CD8^+^ T cells have lost CD62L and acquired CD44 expression; thus showing characteristics of effector/memory T cells.

Taken together, the P → F1 together with congenic markers and proliferation dye labeling provides a valid tool to track proliferation as well as the activation status of allogen-activated T cells on a single cell level, either at a defined endpoint from the spleen or over time from the blood.

### 2.2. Addressing a Hypo-Responsive T Cell Phenotype with the P → F1 Model

**Figure 3:**
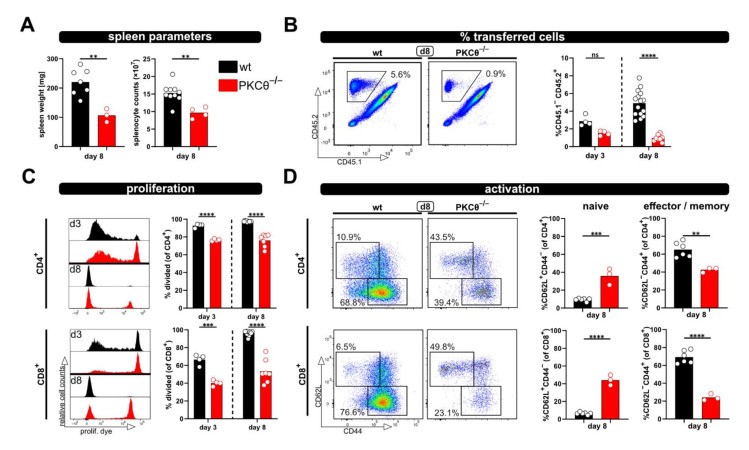
Using PKCθ^-/-^ T cells in the modified P → F1 approach as an example of a hypo-reactive T cell response. (**A**) Spleen weight and splenocyte counts were determined at day 8 after ACT of 8 × 10^6^ wild type or PKCθ-deficient CD3^+^ T cells to allogenic recipients. (**B**,**C**) Flow cytometric analysis of the frequency (**B**) or proliferation (**C**) of either transferred wild type or PKCθ^-/-^ T cells in the spleens of allogenic recipients at day 3 and 8 after ACT. (**D**) Naive and effector/memory subsets of transferred CD4^+^ and CD8^+^ T cells, either wild type or PKCθ^-/-^, were analyzed at day 8 in the spleens of allogenic recipients. Data are represented as individual values plus mean. Representative FACS dot plots or histograms of individual mice are shown. ns = not significant, ** *p* < 0.01, *** *p* < 0.001, **** *p* < 0.0001; (**A**,**D**) unpaired *t*-test; (**B**,**C**) one-way ANOVA following Bonferroni’s post hoc test.

Next, we aimed to test whether the relevance of “genes of interest” for allogenic T cell activation in vivo can be determined by applying the modified P → F1 model. Therefore, T cells from protein kinase C-theta (PKCθ)-deficient mice, a knockout-mouse strain known to have an impaired T cell response, were used. Others, as well as our laboratory, have already demonstrated that PKCθ is activated downstream of the TCR complex and is involved in early T cell activation [11,15]. Thus, the knockout of PKCθ results in a hypo-responsive T cell phenotype with reduced proliferation and IL-2 production [11,15]. As expected, allogenic recipients of PKCθ-deficient T cells demonstrated significantly reduced spleen weight and splenocyte counts compared to recipients of wild type T cells (Figure 3A). Of note, spleen parameters were (almost) comparable to what was observed with syngenic recipients of wild type T cells (Figure 1B). Accordingly, the percentage of transferred PKCθ-deficient T cells was lower than transferred wild type T cells and remained at levels comparable to the percentage of wild type T cells in syngenic recipients (Figure 1C and Figure 3B). The difference between T cells of both genotypes was especially pronounced at day 8, in agreement to what we expected for hypo-responsive T cells. Moreover, significant proliferation deficiency (Figure 3C) and impaired transition from naive to effector/memory T cells (Figure 3D) was observed with PKCθ-deficient CD4^+^ as well as CD8^+^ T cells.

**Figure 4:**
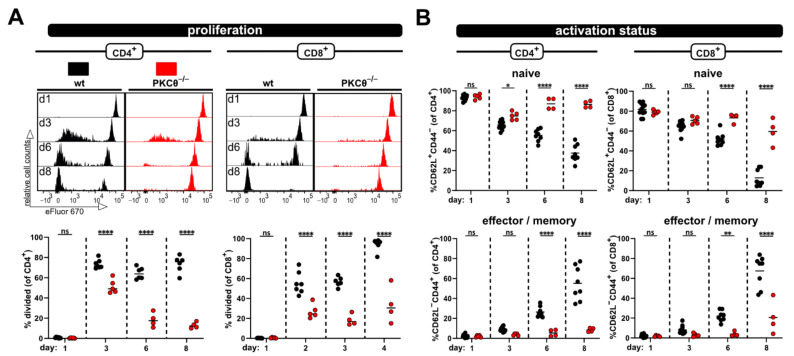
Impaired allogen-induced activation of *PKCθ*-deficient T-cells can be detected in blood. (**A**) Proliferation and (**B**) CD4^+^ and CD8^+^ T cell subsets (naive and effector/memory) were analyzed at the same time points by flow cytometry from blood. Data are represented as individual values plus mean. Data are represented as individual values plus mean. Representative FACS histograms of individual mice are shown. ns = not significant, * *p* < 0.05, ** *p* < 0.01, **** *p* < 0.0001; one-way ANOVA following Bonferroni’s post hoc test.

Furthermore, the hypo-responsive phenotype of PKCθ-deficient T cells was also detected in blood samples taken at several time points after ACT (Figure 4). Initially, at day 3 the proliferation of PKCθ-deficient T cells (especially CD4^+^ T cells) was quite similar to wild type T cells. However, the frequency of divided T cells lacking PKCθ in contrast to wild type T cells did not further increase with time, but rather declined (Figure 4A). The activation defect of PKCθ-deficient T cells was also clearly visible with regard to the impaired acquisition of an effector/memory phenotype over time. While most wild type T cells were CD44^+^CD62L^−^ at day 8, PKCθ-deficient T cells remained CD44^−^CD62L^+^, thus retaining their naive phenotype (Figure 4B).

### 2.3. Adjustment of the P → F1 Model

**Figure 5:**
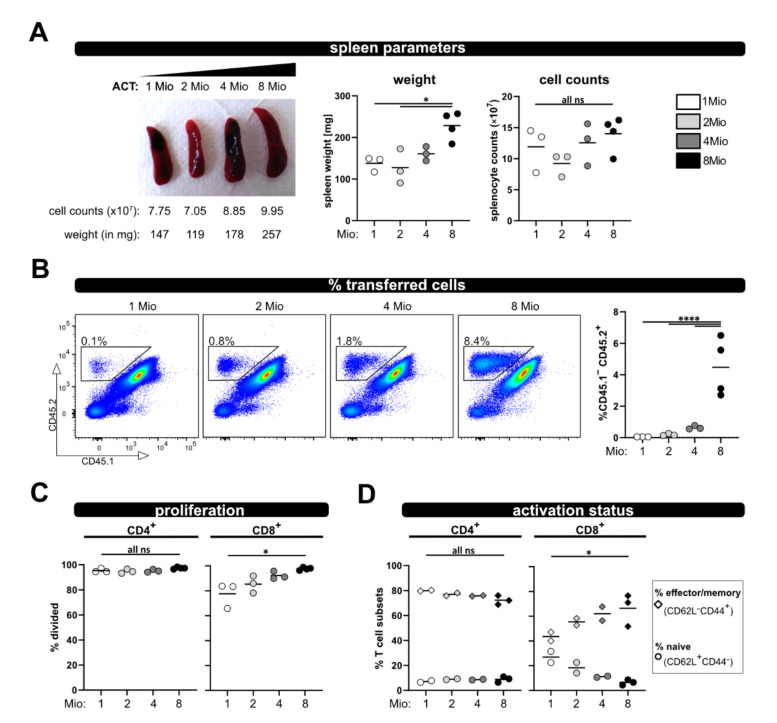
Titration of transferred T cell numbers. (**A**) Representative pictures of spleens on day 8 after transfer of 1, 2, 4 or 8 million wild type T cells are depicted. (**B**) Frequency of the transferred T cells among splenocytes and from blood at day 8 after ACT was determined by flow cytometry. (**C**) Proliferation and (**D**) T cell activation status (naive and effector/memory) of transferred wild type CD4^+^ and CD8^+^ T cells was analyzed at day 8 by CD62L and CD44 staining and flow cytometry. Data are represented as individual values plus mean. Representative FACS dot plots of individual mice are shown. ns = not significant, * *p* < 0.05, **** *p* < 0.0001; one-way ANOVA following Bonferroni’s post hoc test.

To enable analysis in case less T cells (e.g., analyzing subsets) are available for ACT, and also to reduce the number of needed donor mice, we tested four different numbers of transferred T cells (1, 2, 4 and 8 million) and analyzed recipient mice at day 8. The spleen weight was comparable between 1, 2 and 4 million transferred T cells, and only significantly increased when 8 million T cells were transferred (Figure 5A). Additionally, there were no gross differences in spleen counts between all four groups. However, as expected, the less T cells were transferred, the lower was their frequency in the spleen and blood, and only reached significance when compared to 8 million (Figure 5B). Most importantly with all four T cell numbers, vigorous proliferation and acquisition of an effector/memory phenotype was observed (Figure 5C,D). While the allogenic response of CD8^+^ T cells demonstrated some dependency on the transferred T cell number (but only significant for the comparison of 1× and 8 × 10^6^ transferred T cells), the activation and proliferation of CD4^+^ T cells was similar between the four distinct groups, Figure 5C,D). Therefore, we conclude that 1 × 10^6^ transferred T cells are enough to analyze in vivo activation in this allogenic model.

## 3. Discussion

The P → F1 model, established more than 40 years ago [16], has been used in several studies as a disease model to understand T cell regulating factors important for the development of aGVHD or lupus-like cGVHD. Initially, activated donor CD4^+^ T cells expand and provide help for host B cells, which proliferate and produce autoantibodies. In addition, donor CD8^+^ T cells will be activated and differentiate into cytotoxic T lymphocytes (CTL) that eliminate host lymphocytes, primarily B cells. The latter response leads to aGVHD with a profound immunodeficiency by day 14, counteracting donor-CD4^+^/host-B cell-mediated autoimmune symptoms (reviewed in [13]). Of note, the choice of the parental donor determines the disease phenotype. For example, while a transfer of T cells from a C57BL/6 donor to a B6D2F1 recipient (B6 → F1) results in an aGVHD phenotype, the transfer of DBA-donor T cells (DBA → F1) induces a lupus-like cGVHD. This might be due to the differences in the strain-specific quality of CD8^+^ CTL responses. For instance, for the C57BL/6 mouse strain, in contrast to DBA mice, high proportions of cytotoxic CD8^+^ T cells have been described [17]. Furthermore, CD4^+^ T cells of DBA mice have been demonstrated to be poor IL-2 producers and thus provide less help for CD8^+^ T cells [18]. Altogether, these studies highlight the importance of fine-tuning effector T cell responses of the distinct T cell subpopulations to impact disease outcome. In this regard, if CD4^+^ T cells are transferred to F1-hosts without donor CD8^+^ T cells, a lupus-like cGVHD develops instead of aGVHD [19]. Hence, under these circumstances, donor CD8^+^ CTL responses are beneficial, since they counteract autoantibody production by killing host B cells.

Of note, using the P → F1 model, different approaches have been conducted to modulate T cell responses and thereby affect the disease outcome to induce either acute or chronic GVHD. In brief, while the blocking of CTLA-4 simultaneous with ACT prevented the development of acute and chronic GVHD, other treatments promoted either one or the other disease phenotype [20]. Treatment of mice with an agonistic anti-CD40 antibody, an antibody blocking CD80, recombinant IL-12 or IL-21 promotes CTL-development and leads to aGVHD in the DBA → F1 model, which usually develop cGVHD [21,22,23,24]. In contrast, the transfer of IL-21R-deficient donor T cells, early neutralization of TNF-α, or administration of IL-233, a hybrid cytokine composed of IL-2 and IL-33, impaired the donor CD8^+^ T cell expansion and maturation, thereby attenuating aGVHD symptoms in the B6 → F1 model [22,25,26].

The mentioned findings focus primarily on the outcome of the two major P → F1 disease phenotypes, which can be mainly distinguished by the extent of donor T cell engraftment, elimination or expansion of host B cells and serum anti-DNA antibody levels, at later time points (10 days to months). However, we are interested in rather early events of T cell activation on a cellular and molecular level. Thus, we modified the P → F1 model to assess how genetic alterations influence T cell activation by analyzing the phenotype of T cells from knockout mice within 8 days after transfer. Using this time frame for our analyses, no massive killing of endogenous cells and also no severe health issues are expected from what is known from earlier studies [13]. This model provides an in vivo T cell stimulation without the need of irradiation or lymphodepletion of the recipients. Of note, in contrast to many other studies applying the P → F1 model, we only transfer donor T cells instead of huge numbers of unfractionated parental strain splenocytes (typically 50 × 10^6^ C67BL/6 donor T cells are used [27]). Furthermore, since we use CD45 isoforms to distinguish donor and host T cells, similar to what has been performed by Suchin et al., we are able to track transferred T cells and determine their proliferation even when the proliferation dye labeling has already been diluted to unstained levels [9,28]. Besides endpoint analyses addressing the splenic T cell subsets, it is also possible to follow the donor T cell phenotypes over time by taking blood samples in a minimal invasive way. Of note, the frequency of transferred T cells was lower at day 6 in blood than at day 3 or day 8. We can only speculate why this is the case: the trapping of the allogenic T cells in organs, where they recognize their cognate antigen and also re-distribution to peripheral tissues, which is a characteristic of effector T cells, might contribute.

We believe that this model based on transferring congenic allo-reactive T cells is a valuable tool for the investigation of genes of interest regarding a physiological T cell activation and effector function in vivo. In addition to the here described readouts, it is also possible to investigate tissue localization of the transferred T cells, for example by immunohistochemistry, and tissue infiltrating cells, e.g., from the gut, could be analyzed by flow cytometry.

Using this approach, we observed enlarged spleens as well as increased splenocyte counts on day 3 and 8 after ACT, accompanied by the massive proliferation of CD4^+^ as well as CD8^+^ T cells in allogenic but not syngenic recipients. In this regard, the flow cytometric analysis revealed that the donor T cell phenotype changes from a dominantly naive to an exclusively effector/memory state and acquires exhausted profiles on day 8 after ACT. This can also be observed in a blood kinetic over time (d1, d3, d6, d8) with the accumulation of proliferated cells and a switch from a naive to an effector phenotype. However, this was all impaired in the case of PKCθ-deficient donor T cells, supporting our attempt to use this model in the future to determine genes that might be positive regulators of T cell functions. Our results, demonstrating reduced allogenic proliferation of T cells lacking PKCθ, are in line with what is known from the literature implicating a role of PKCθ in alloreactivity and GVHD. Thus, PKCθ-deficient donor T cells demonstrated reduced ability to induce disease in MHC-mismatched hosts (using TCD-BMT GVHD models [29,30], fitting to impaired in vivo proliferation in allogenic settings at day 3 or 4 after ACT [29,31]. Additionally, inhibitors for PKCθ, but also targeting PKCα, could prevent GVHD [30].

Furthermore, we could demonstrate that downscaling the number of transferred T cells is possible. In this case, it is feasible to use the model if less cells are available; we tested as few as one million transferred T cells. However, one has to be aware that decreasing numbers of transferred T cells might impede the analysis. In particular, the tracking of parameters over time in blood samples will be difficult, since there is only limited availability of blood, which can be harvested per time point from a living animal. Moreover, we observed that CD8^+^ T cell activation was slightly stronger the more T cells have been transferred. Therefore, it is obviously important that equal T cell numbers are transferred; especially when comparing wild type and knockout T cells. In this regard, it has been demonstrated that the transfer of suboptimal donor T cells numbers (in their study ≤ 30 × 10^6^ unfractionated splenocytes) does not induce aGVHD [27]. Nevertheless, we could demonstrate that it is possible to analyze the allogenic response on a single cell level even if reduced T cell numbers are used for ACT. Thus, the model could be also extended to investigations of T cell subpopulations, e.g., transferring CD4^+^ or CD8^+^ T cells separately or performing a transfer of genetically modified CD4^+^ T cells together with wild type CD8^+^ T cells, or the other way around, depending on the research question.

Taken together, our findings prove that the here described model is well suited to investigate regulators of T cell responses at a single cell level in vivo. Here, we focused on a basic setting, where genetically modified T cells are transferred to allogenic recipients serving as a starting point for a highly flexible, physiological model system investigating T cell activation in vivo, which can be adapted to one’s specific research questions and technical possibilities.

## 4. Materials and Methods

### 4.1. Mice

Male DBA/2J mice were purchased from The Jackson Laboratory (strain #000671) and crossed with B6.SJL-Ptprca Pepcb/BoyJ (strain #002014; “CD45.1^+^/1^+^”) females to receive a hybrid (F1 generation; B6D2F1). Syngenic controls were obtained by the breeding of C57BL/6J (strain #000664) to B6.SJL-Ptprca Pepcb/BoyJ mice. PKCθ-deficient mice ([11]) were used to investigate hypo-responses. Mice were maintained under specific pathogen-free (SPF) conditions. All animal experiments were performed in accordance with national and European guidelines and authorized by the committee on animal experiments (2020-0.345.526 and BMWFW-66.011/0076-WF/V/3b/2018). Experimental mice were chosen randomly from litters.

### 4.2. Splenocyte Isolation, T Cell Sorting and Proliferation Dye Labeling

Single cell suspensions of spleens were prepared by mechanical disintegration using metal sieves or 100 µM cell strainers (Falcon). Thereafter, erythrocytes were removed by lysis (0.15 M NH_4_Cl, 10 mM KHCO_3_ and 0.1 mM EDTA) for 2-6 min at room temperature (RT), followed by a wash/ filter step using 1× PBS, 0.5% BSA, 0.5 M EDTA and 40 µM cell strainers (Falcon). Viable cell counts were determined after staining with acridine orange and propidium iodide (AO/PI) Cell Viability Kit (F23001-LG, Biocat, Heidelberg, Germany) on a LUNA Automated Cell Counter (Logos Biosystems, Villeneuve d’Ascq, France).

CD3^+^ T cells were sorted untouched by negative selection using MACS technology-based isolation kits (130-095-130, pan-T cells) along with pre-separation filters, LS columns and a QuadroMACS separator (all Miltenyi Biotec, Bergisch Gladbach, Germany), according to the manufacturer’s instructions. Sort purity was checked by flow cytometry.

Isolated CD3^+^ T cells were labeled with cell proliferation dye eFluor670 (ebiosciences 65-0840). Therefore, a twofold labeling solution (10 µM) was prepared in HBSS with Mg^2+^ and Ca^2+^ (HBSS++). T cells were washed twice with HBSS++ at RT and adjusted to 1 × 10^7^ cells/mL. An equal volume of the twofold labeling solution was added and mixed immediately with the cells by inverting the tube. Cells were incubated for 5 min at 37 °C in the dark. The reaction was stopped with cold FCS and RPMI followed by a wash step with RPMI and subsequently with HBSS++. Finally, cells were resuspended in HBSS++ (8 × 10^6^/100 µL) and injected intravenously.

### 4.3. P → F1 Model

CD3^+^ T cells from C57BL/6 mice (CD45.2, MHC haplotype b) were isolated as described above, labeled with proliferation dye and transferred to allogenic B6D2F1 (MHC haplotype b/d) or syngeneic control (MHC haplotype b) recipients. A total of 1, 2, 4 or 8 × 10^6^ T cells resuspended in 100 µL HBSS++ were injected into the lateral tail vein. For endpoint analyses, spleens from recipient mice were harvested and weighted on day 3 or day 8 after ACT. Total splenocyte counts were determined from single cell suspension after the lysis of erythrocytes. For T cell analyses from blood, the mice were bled from the facial vein using a lancet into tubes containing heparin. Subsequently, splenocytes or blood samples (after lysis of erythrocytes) were analyzed by flow cytometry.

### 4.4. Flow Cytometry

Flow cytometric analyses were performed on FACS Canto II (4-2-2 configuration, BD Biosciences, Franklin Lakes, NJ, USA) and the subsequent data processing was conducted with the FlowJo software 10.8.0 (BD, Ashland, OR, USA). For surface stainings, the cells were incubated for 5 min with FcR-block (anti-CD16/32; BD Biosciences) prior to the addition of the antibody mix in PBS, 0.5% BSA, 0.5 M EDTA. Subsequently, the cells were incubated for 20 min with the antibody solution. Lastly, the cells were washed with PBS, 0.5% BSA, 0.5 M EDTA and transferred into FACS tubes (all steps at 4 °C). The following antibodies were used for surface staining: CD4 V500 (clone RM4-5; BioLegend, San Diego, CA, USA), CD8 PerCPCy5.5, CD8 BV510, CD8 APC-Cy7 (all clone 53-6.7; BioLegend), CD44 PE-Cy7, CD44 FITC (both clone IM7; BioLegend), CD45.1 PB, CD45.1 APC-Cy7 (both clone A20; BioLegend), CD45.2 FITC, CD45.2 PE-Cy7 (both clone 104; BioLegend), CD45.2 V500 (clone 104; BD Biosciences) and CD62L APC-Cy7 (clone MEL-14; BioLegend). To exclude dead cells, the cells were stained with the Fixable Viability Stain 780 (BD Biosciences, diluted 1:2000 in HBSS++) for 10 min at RT in the dark according to the manufacturer’s instructions. The following gating strategy was applied: (1) exclusion of dead cells via viability staining, (2) gating on lymphocytes in the FSC/SSC plot, (3) double cell exclusion, (4) gating on transferred cells via CD45.1/2 and (5) CD4 or CD8 blotted against proliferation dye and (6) gating on the parameter of interest (proliferation dye, CD44 vs. CD62L).

### 4.5. Statistical Analysis

Data were analyzed using the GraphPad Prism 9.0.1 software (GraphPad Software, San Diego, CA, USA). The data were analyzed for statistical significance by one-way ANOVA following Bonferroni’s post hoc test (for comparisons on more time points or more groups) or by the Student’s *t*-test (for simple comparisons between two groups), as indicated in the figure legends. A p-value of <0.05 was considered statistically significant. Symbols used in the figures are: * *p* < 0.05; ** *p* < 0.01; *** *p* < 0.001; **** *p* < 0.0001.

## Data Availability

Not applicable.

## Supplementary Materials

The following supporting information can be downloaded at: https://www.mdpi.com/article/10.3390/ijms23105337/s1.
