# Supplementary material for: A MLR-Based Approach to Analyze Regulators of T Lymphocyte Activation In Vivo
Source: Int J Mol Sci. 2022 May 10;23(10):5337. doi: 10.3390/ijms23105337 (PMC9140849; doi:10.3390/ijms23105337)
Supplement: Supplementary file 1 [file ijms-23-05337-s001.zip › ijms-1627227-supplementary.pdf]

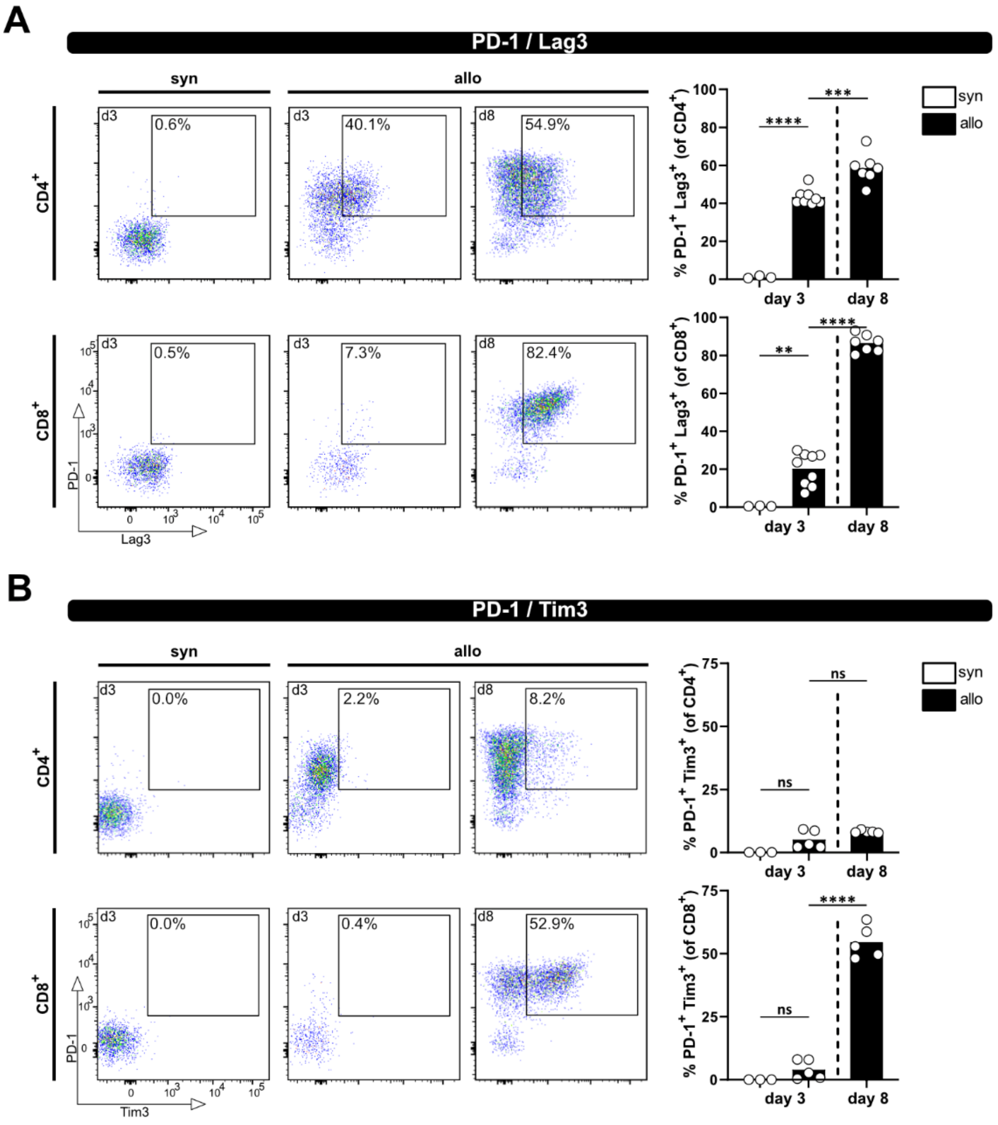

Supplementary Figure S1. Chronically activated allogenic T cells up-regulate inhibitory receptors.
The frequency of PD-1$^+$Lag3$^+$ (**a**) and PD-1$^+$Tim3$^+$ (**b**) cells among transferred CD4$^+$ and CD8$^+$ T cells was analyzed by flow cytometry in syngenic (day 3 of ACT) and allogenic (day 3 and day 8) recipients. The experimental setup is the same as described in Figure 1. Data are represented as individual values plus mean. Representative FACS dot plots of individual mice are shown. ns = not significant, ** $p < 0.01$, *** $p < 0.001$, **** $p < 0.0001$; unpaired t-test.
